# Lysine specific demethylase 1 is a molecular driver and therapeutic target in sarcoma

**DOI:** 10.3389/fonc.2022.1076581

**Published:** 2023-01-04

**Authors:** Rachel D. Dreher, Emily R. Theisen

**Affiliations:** ^1^ Abigail Wexner Research Institute, Center for Childhood Cancer and Blood Diseases, Nationwide Children’s Hospital, Columbus, OH, United States; ^2^ Biomedical Sciences Graduate Program, College of Medicine, the Ohio State University, Columbus, OH, United States; ^3^ Department of Pediatrics, College of Medicine, The Ohio State University, Columbus, OH, United States

**Keywords:** LSD1, sarcoma, mesenchymal development, oncogenic fusion protein, epigenetics

## Abstract

Sarcomas are a diverse group of tumors with numerous oncogenic drivers, and display varied clinical behaviors and prognoses. This complexity makes diagnosis and the development of new and effective treatments challenging. An incomplete understanding of both cell of origin and the biological drivers of sarcomas complicates efforts to develop clinically relevant model systems and find new molecular targets. Notably, the histone lysine specific demethylase 1 (LSD1) is overexpressed in a number of different sarcomas and is a potential therapeutic target in these malignancies. With the ability to modify histone marks, LSD1 is a key player in many protein complexes that epigenetically regulate gene expression. It is a largely context dependent enzyme, having vastly different and often opposing roles depending on the cellular environment and which interaction partners are involved. LSD1 has been implicated in the development of many different types of cancer, but its role in bone and soft tissue sarcomas remains poorly understood. In this review, we compiled what is known about the LSD1 function in various sarcomas, to determine where knowledge is lacking and to find what theme emerge to characterize how LSD1 is a key molecular driver in bone and soft tissue sarcoma. We further discuss the current clinical landscape for the development of LSD1 inhibitors and where sarcomas have been included in early clinical trials.

## 1 Challenges in sarcoma diagnosis and treatment

Sarcomas are a diverse group of tumors. Arising from a mesenchymal origin, sarcomas present in bones and soft tissues. There are currently over 100 distinct sarcoma subtypes based on the histological phenotype of the tumor. In the broadest terms, these subtypes can be categorized into two overarching groups; the more common soft tissue sarcomas and primary bone sarcomas (1). Soft tissue sarcomas make up less than 1% of all malignancies, and typically arise in skin, organs, or other soft tissues that include fat, muscle, nerve sheath, blood vessels (2). Osteosarcoma is the most prevalent primary bone cancer in children, and only makes up approximately 5% of all childhood cancers (1). Overall, soft tissue sarcomas have an estimated annual incidence of 4.74 out of 100,000 individuals and bone sarcomas are 0.8 out of 100,000 individuals (3).

The diversity of sarcoma types presents a challenge to both diagnosis and treatment. In addition to the numerous types of sarcoma, there are frequently unusual presentations within each subtype (4). The same type of sarcoma can present in varied organs with vastly different clinical behavior, and there are cases when a tumor does not adhere to any of the diagnostic criteria (4). Unlike more common hematological and epithelial malignancies, which usually arise from dedifferentiation of a benign precursor, the majority of sarcomas are sporadic and idiopathic, with unknown cells of origin (5). Some sarcomas harbor a single genetic defect, such as a chromosomal translocation. Others contain multiple complex genetic defects, such as mutations in cell cycle genes causing genomic instability (6). Sarcomas can also arise as part of a genetic syndrome, most commonly Li Fraumeni Syndrome or Neurofibromatosis 1 (6). Further complicating diagnosis is the scarcity of sarcoma patients. Clinicians frequently do not see enough of these rare tumors to develop the expertise required for accurate diagnoses. In fact, sarcoma diagnosis is one of the most challenging aspects of diagnostic pathology and misdiagnoses occur in 20-30% of cases (7).

Once diagnosed, the standard of care for sarcomas includes surgery, chemotherapy, and/or radiotherapy (1). These treatment regimens have shown improvement over time, but remain futile against relapsed or refractory sarcomas and often come with serious side effects (8). There is an urgent need to identify and develop new therapeutic strategies with improved potency and safety profiles. Therapies targeting the molecular drivers of sarcomas have the potential to improve patient outcomes with decreased side effects. However, target identification in sarcomas is hindered by our poor understanding of the basic mechanisms underlying the biology of these tumors. Moreover, the diversity of sarcomas and the relatively low numbers of patients for each type of sarcoma hinders the research that could lead to these types of treatments. The identification of molecular drivers that are common to multiple subtypes are therefore attractive targets. In this review, we will discuss one such target, the histone lysine specific demethylase 1 (LSD1).

### 1.1 LSD1 is overexpressed in various sarcomas

LSD1 emerged as a possible therapeutic target for sarcomas in the early 2010s. Early studies demonstrated that LSD1 regulates broad changes in gene expression and is involved in tumor progression in multiple tumor types (9–11). With this rationale, in 2011 Schildhaus et al. sought to extend these findings to sarcoma and screened 468 mesenchymal tumors, ranging from benign to highly malignant, for LSD1 expression levels (12). A year later, Bannani-Baiti et al. screened a second panel of mesenchymal tumors for LSD1 expression levels (13). These two studies identified LSD1 overexpression in solitary fibrous tumors, synovial sarcoma, rhabdomyosarcoma, desmoplastic small round cell tumors, malignant peripheral nerve sheath tumors (12), and osteosarcoma, Ewing sarcoma, and chondrosarcoma, among others (13). Of note, other histone demethylase proteins, such as JARID1C and JMJD2C, were not overexpressed in these mesenchymal tumors. Therefore, any excess demethylase activity is a direct result of aberrant LSD1 expression (13).

LSD1 is a promising therapeutic target for a few key reasons. First, LSD1 is pharmacologically targetable, and several classes of small molecule inhibitors targeting both enzymatic and nonenzymatic LSD1 activity have been developed. Additionally, patient prognosis is inversely correlated with LSD1 expression (14). Excess LSD1 activity resulting from overexpression may play a critical role in aggressive tumor biology and targeting it may improve patient outcomes. There are few reported somatic mutations in the *KDM1A* gene encoding LSD1 in cancer and LSD1 localizes to the nucleus in sarcomas, together indicating that it continues to perform its native function when overexpressed (13). This also suggests that the likelihood of developed resistance to targeted inhibition is lower (14). More work is required to fully understand the functional consequences of LSD1 overexpression and how this contributes to prognosis, particularly in light of the recent data suggesting that LSD1 possesses both enzymatic and nonenzymatic activity (15, 16). In this review we introduce LSD1 and provide an overview of LSD1 containing complexes. We will then discuss how aberrant LSD1 activity drives the oncogenic process in specific sarcomas, and conclude with a discussion on the current clinical approaches for targeting LSD1.

## 2 Discovery of LSD1

Prior to the discovery of LSD1, histone methylation was considered a static process. Unlike the dynamic regulation of histone acetylation, through which acetyl groups are deposited by histone acetyltransferases (HATs) and removed by histone deacetylases (HDACs), there was no known mechanism to actively remove methylated histone residues (17). Shi et al. proposed that the only way to remove a methylated histone residue would be to “clip” the methylated histone tail or to replace the entire histone protein, both inefficient processes (17).

This all changed in 2003 when Shi et al. identified a novel nuclear polyamine oxidase protein, which they referred to as KIAA0601 (17). KIAA0601 was discovered in the C terminal Binding Protein (CtBP) corepressor complex, a complex known to regulate gene expression *via* histone modifying events (18). Conserved throughout eukaryotes, KIAA0601 homologs were shown to repress transcription in coordination with the CtBP corepressor complex (17). Subsequent work from the Shi et al. group revealed that KIAA0601 functioned as a corepressor by specifically removing the methylation marks from lysine 4 on the histone H3 tail (17). As such, they renamed the protein to reflect its function; lysine specific demethylase 1 (LSD1) (17).

### 2.1 Structure and catalytic activity of LSD1

**Figure 1:**
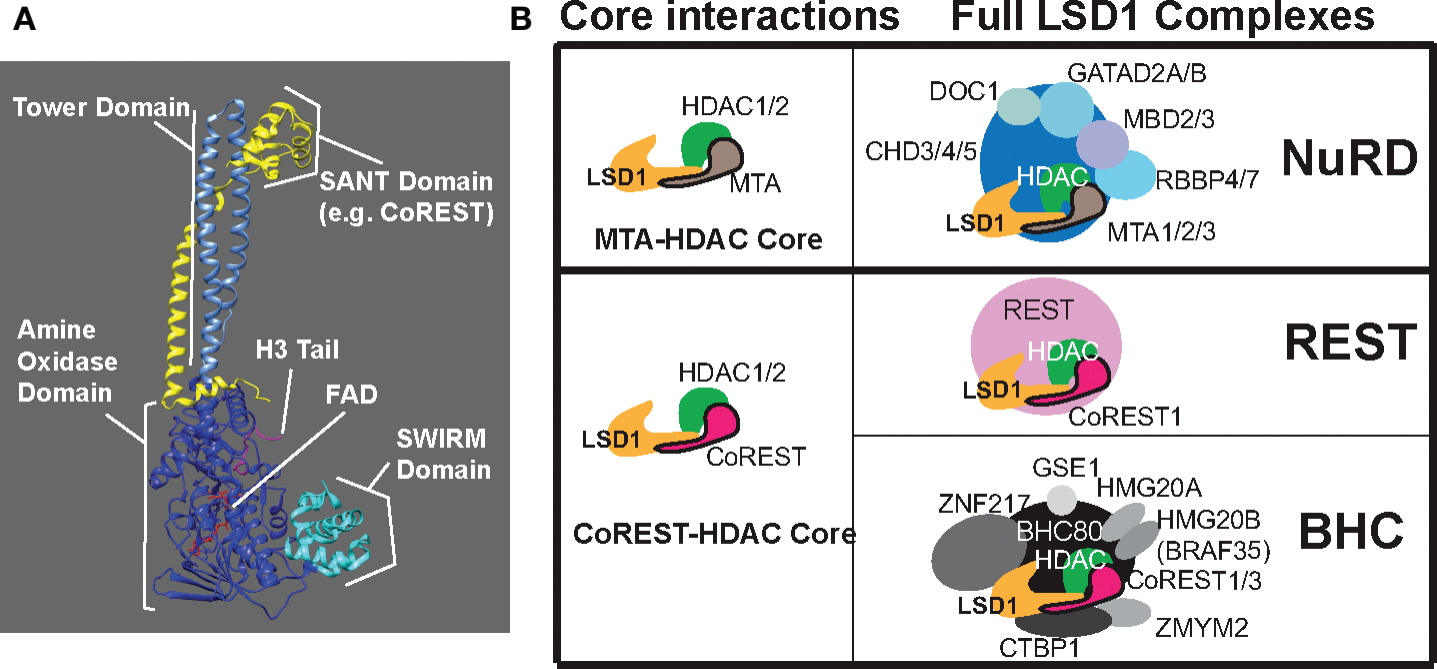
LSD1 structure and LSD1 containing complexes. **(A)** Structural illustration of LSD1 in complex with a SANT domain of CoREST or MTA bound to histone H3 tail substrate and cofactor FAD. Essential domains of LSD1 are labeled; tower domain, amine oxidase domain, and the SWIRM domain. **(B)** LSD1 containing complexes broken down by core interactions and example full complexes.

LSD1 has three main domains forming the core of the protein; an N-terminal SWIRM domain, a bi-lobed C-terminal amine oxidase domain (AOD), and a tower domain that protrudes from the AOD (19)(**Figure 1A**). The SWIRM domain, also found in other histone modifying proteins, facilitates protein-protein interactions with the histone H3 N-terminal tail (19). One lobe of the AOD mediates flavin adenine dinucleotide (FAD) cofactor binding and the other lobe is important for substrate recognition (19). The active site of LSD1 is much larger than other amine oxidase proteins such as monoamine oxidase A or B (MAO-A/B), which allows for binding of the larger histone H3 tail (19). The tower domain, consisting of two long, antiparallel, non-coiling alpha helices (**Figure 1A**), is unique to LSD1, and is critical for the inclusion of LSD1 into chromatin regulatory complexes (19). LSD1 lacks inherent DNA binding activity, so its inclusion in these complexes allows LSD1 to access nucleosomal substrates in the cell (19).

LSD1 removes methyl groups from lysine residues on the histone H3 tail through an FAD-dependent oxidative reaction (20). Each H3 subunit has multiple lysine residues along the tail that can be methylated once (monomethyl, me1), twice (dimethyl, me2), or three times (trimethyl, me3) (17). LSD1 can distinguish between different levels of methylation on a singular lysine residue, and between the same level of methylation on different lysine residues (17). Interestingly, LSD1 removes both the mono- and dimethyl marks, but is unable to demethylate trimethyl lysine residues. This is due to the chemical constraints of the oxidative demethylation mechanism (20). A methylated lysine residue is oxidized *via* the cofactor FAD, which forms hydrogen peroxide and an intermediate imine. This imine is then hydrolyzed, producing the demethylated lysine residue and formaldehyde. Trimethyllysine residues are unable to form the intermediate imine and therefore cannot be demethylated *via* this reaction sequence. As such, LSD1is limited to demethylation of mono- and dimethyllysine (20).

LSD1 removes mono- and dimethyl residues from lysine 4 (H3K4) and lysine 9 (H3K9) (21). Methylation of H3K4 is an activating mark, while methylation of H3K9 is a repressive mark. As such, LSD1 function can either be repressive or activating, depending on its target. Several factors determine the catalytic efficiency and specificity of LSD1. The first 20 N terminal amino acids of the H3 histone tail are highly charged and are essential for recognition (21). LSD1 is sensitive to other posttranslational modifications on the histone H3 tail. Some of these, such as acetylation at H3K9 (H3K9ac), can diminish LSD1 activity toward H3K4. Others, such as phosphorylation at serine 6 (H3S6), can promote LSD1 specificity for H3K9. Alternatively, phosphorylation of serine 10 (H3S10) blocks LSD1 activity at H3K9. By reading the histone tail in such a manner, LSD1 requires the activity of other enzymes, such as phosphatases and deacetylases to remove other marks first (21). Supporting this Forneris et al. showed that LSD1 is most efficient at demethylating H3K4 when there are no other PTMs on the histone tail (21).

### 2.2 LSD1 in complexes

Another factor that determines substrate specificity, and therefore biological function, is the complex in which LSD1 resides (22, 23). For example, early studies showed LSD1 interacts with androgen and estrogen receptors, and that these interactions promote LSD1-mediated demethylation of H3K9 (9, 24). In other complexes, such as the co-repressor of repressor element-1 silencing transcription factor (CoREST) complex, LSD1 targets H3K4me for demethylation (22). Different LSD1 complexes have been identified in different cell types and different contexts (25–27). Here we’ll briefly discuss the more commonly observed LSD1 complex composition, with a focus on those complexes likely to be important in sarcoma.

### 2.2.1 LSD1 in CoREST complexes

LSD1-CoREST complexes are the most studied LSD1-containing complexes. These complexes are most often comprised of a core containing LSD1, CoREST, and HDAC1 or HDAC2 (22). The original description of the CoREST complex included the transcriptional repressor REST protein as an associated complex member (28) (**Figure 1B**). Perhaps the most common interaction partner for LSD1 is CoREST, which binds to the tower domain of LSD1. CoREST proteins contain one ELM2 domain and two SANT domains, SANT1 and SANT2 (29). ELM2 and SANT1 both interact with HDACs, while the SANT2 domain interacts with LSD1 (29). The CoREST complex places LSD1 in the correct position for demethylation with various interactions tethering LSD1-CoREST to the substrate on the H3 histone tail (28). Once the proper contacts have been made, the C-terminal region of CoREST protects LSD1 from degradation and stimulates LSD1 activity (30).

There are three known isoforms of the CoREST protein (CoREST1, CoREST2, and CoREST3) that interact with LSD1 (31). Multiple isoforms of a protein usually suggest some divergent functions, and in this case these differing isoforms regulate the overall function of the LSD1-CoREST complex. Upadhyay et al. determined CoREST1 is the predominant isoform incorporated into this complex, and most studies primarily consider LSD1-CoREST1 complexes (31). The functional consequences of CoREST2 and CoREST3 are less understood. Upadhyay et al. present evidence that CoREST2 serves a similar purpose to CoREST1, albeit less efficiently, while CoREST3 inhibits LSD1 activity (31). However, Barrios et al. suggest that CoREST1 and CoREST3 are redundant and both enable LSD1 demethylation, while CoREST2 serves an unknown purpose (29). Additionally, CoREST2 has been shown to be predominantly expressed in embryonic stem cells, regulates pluripotency and neurogenesis, and has unique localization to nuclear speckles, suggesting distinct functions for this isoform (32, 33).

An additional layer of regulatory complexity exists in that the LSD1-CoREST-HDAC core can be incorporated into multiple protein regulatory complexes. LSD1 was originally discovered as part of the C terminal binding protein complex, that normally includes LSD1, HDAC1/2, CoREST1, EHMT1/2, and CTBP1 (17). LSD1-CtBP cannot bind DNA itself, and requires the DNA binding zinc finger protein, ZNF516, to facilitate the LSD1-CtBP interaction and repress downstream gene programs (34). This complex has been shown to repress epidermal growth factor receptor (EGFR) in breast cancer cell lines, resulting in decreased cell proliferation and motility (34). In another case, the LSD1-CtBP complex associates with a member of the malignant brain tumor domain containing chromatin readers, SFMBT1, to repress transcription of myogenic differentiation in order to maintain an undifferentiated state of myogenic progenitor cells (35).

Another commonly studied LSD1 containing complex is the BRAF histone deacetylase complex (BHC). This complex includes the same LSD1-CoREST-HDAC1/2 core, and associates with other members including HMG20A/B, PHF21A/BHC80, ZNF217, BRAF35, GSE1, and ZMYM2 (36)(**Figure 1B**). The BHC complex is tightly regulated, but again the details of this regulatory mechanism are conflicting in different studies. In 2005, Shi et al. reported that BHC complex member BHC80, a plant homeodomain finger containing protein, negatively regulates LSD1 demethylase activity (30). Two years later, Lan et al. found evidence that BHC80 actually stabilized the LSD1 complex at its target promoters by binding directly to unmethylated H3K4, thus promoting LSD1 activity (11). Nonetheless, the BHC complex plays important biological roles through association with transcription factors that mediate targeting and repression. For example, LSD1 recruitment by Growth Factor Independence 1B (GFI1B) facilitates the recruitment of BHC members to repress the expression of erythroid lineage specific pluripotency genes to trigger erythroid differentiation (36). The same LSD1-CoREST core associates various distinct complexes to carry out distinct functions.

### 2.2.2 LSD1 in MTA complexes

Though LSD1 is most commonly found in complexes containing CoREST, the tower domain of LSD1 also binds to the metastasis associated proteins (MTAs), which possess a similar SANT2 domain as CoREST (37). This interaction with MTA allows LSD1 to associate with other complexes that regulate a different set of genomic targets. Most prominent among these complexes is the nucleosomal remodeling and deacetylase (NuRD) complex. NuRD complexes contain a chromodomain helicase protein (CHD3, CHD4, or CHD5) with ATP-dependent chromatin remodeling activity, as well as MBD2 or MBD3, RBBP4/7, GATAD2A/B, DOC1, HDAC1/2, MTA1/2/3, and, substochiometrically, LSD1 (37) (**Figure 1B**). Like LSD1, NuRD plays many roles in the cell and regulates differentiation, gene expression, and genome stability (reviewed elsewhere (38)).

Similar to the three CoREST isoforms, the three different MTA isoforms (MTA1, MTA2, or MTA3) of the NuRD complex slightly alter the function of the overall complex. Each isoform is involved in regulating separate, but overlapping, gene sets (37). Interestingly, MTA1 has a unique function involving LSD1. The NuRD complex has a sister complex that functions as a coactivator, called the nucleosomal remodeling factor complex (NURF) (37). Nair et al. found evidence that MTA1 acts as the switch between NuRD and NURF. In order to recruit the NuRD complex for gene repression, methylated MTA1 binds chromatin, H3K9 is methylated, and then MTA1 recruits the components of the NuRD complex (37). In contrast, demethylation of MTA1 by LSD1 destabilizes the NuRD complex, and LSD1 is targeted to demethylate H3K9 (37). The demethylated MTA1 then recruits the components of the NURF complex initiating downstream gene activation (37). Notably, LSD1 demethylates MTA1, a non-histone protein, in this mechanism. This is not the only non-histone protein that is demethylated by LSD1, but these substrates will not be thoroughly discusses as they have been reviewed elsewhere (39).

### 2.2.3 LSD1 in other complexes

Though more thoroughly studied, the complexes discussed above are not the only reported LSD1 complexes. The complete suite of LSD1 complexes is reviewed elsewhere (40). To briefly touch on some of the other reported complexes, we will focus on interactions that promote LSD1 demethylation of H3K9. The most thoroughly studied examples of LSD1 mediated gene activation through activity at H3K9 include interactions with the androgen and estrogen nuclear receptors. LSD1 demethylates H3K9me1/2 to promote androgen and estrogen receptor target gene expression (21, 40). In breast cancer, the nuclear orphan estrogen related receptor α (ERRα) directs LSD1 toward H3K9, activating a transcriptional program that allows cells to invade the extracellular matrix, important in cancer metastasis (24). LSD1 can also demethylate H3K9 to activate gene expression patterns important for neuronal development. This process involves a neuronal specific splice variant of LSD1 that binds to SVIL through new residues included at the base of the tower domain (41). This interaction helps LSD1 recognize H3K9 as its substrate.

LSD1 is a multi-faceted enzyme whose function greatly depends on the complexes it interacts with and the cellular context. Efforts to understand LSD1 function in sarcoma require careful attention to these contextual factors. Importantly, LSD1 has recently been shown to possess nonenzymatic function, and may function primarily as a scaffolding protein in some cases (15, 16). This elevates the importance of understanding the LSD1 interactome in any given cell and tumor type, and determining both which and how LSD1 complexes meaningfully contribute to tumor biology.

## 3 LSD1 in mesenchymal differentiation and development

By acting through various complexes to regulate transcriptional programs, LSD1 is a crucial regulator of cellular differentiation and identity. The biological role for LSD1 changes dynamically from embryonic stem cells through differentiation, with different activities in different lineages. In embryonic stem cells, the LSD1-CoREST complex plays important roles suppressing differentiation and promoting gene expression programs important for the maintenance of pluripotency (42). However, once a cell begins to differentiate, the LSD1-NuRD complex decommissions the enhancers important for pluripotency to facilitate differentiation (43).

LSD1 is known to play important roles in hematopoietic and neuronal differentiation, where it suppresses expression of neuronal genes in non-neuronal lineages (reviewed elsewhere (41, 44)). However, the role that LSD1 plays in normal mesenchymal differentiation is less studied, though there are some descriptions of LSD1 activity in myogenesis, osteogenesis, and adipogenesis. There are no reports of LSD1 function in chondrogenesis. Expression of LSD1 is important for the maintenance of mesenchymal stem cell gene expression programs, and multiple methods to block LSD1 activity induce the differentiation of mesenchymal stem cells (MSC) (45, 46). There is also evidence that LSD1 is a critical regulator in determining whether a cell progresses down a myogenic or osteogenic differentiation pathway. LSD1 was shown to be the only lysine demethylase important for myogenesis and represses the expression of the osteogenic master regulator RUNX2 in C2C12 cell (47).

### 3.1 Myogenesis

In myogenesis, LSD1 physically interacts with myogenic factors MyoD and Mef2 to localize to myoblastic differentiation genes. Here LSD1 removes the repressive histone methylation signature and subsequently induces skeletal muscle differentiation (48). LSD1 also demethylates a non-histone substrate, transcription factor Mef2d, which increases its activity and in turn upregulates myogenic genes in muscle cell differentiation (49). Furthermore, LSD1 is deeply influenced by the metabolic glucocorticoid environment during myogenesis (50). Lower levels of glucocorticoids result in reduced degradation of LSD1, allowing LSD1 to repress oxidative metabolism and slow-twitch myosin genes (50).

### 3.2 Osteogenesis

In osteogenesis, the work that has been done demonstrates opposing functions of LSD1 in regulating bone formation. LSD1 is highly expressed in osteoblasts and is recruited to specific DNA motifs at osteogenic promoters to remove repressive histone methylation marks (25). LSD1 activity thus induces differentiation of osteoblasts to promote bone formation and *in vitro* knockdown of LSD1 in mouse mesenchymal cells impaired normal bone formation (25). Similarly, in fracture repair LSD1 represses retinoic acid signaling in order to allow cartilaginous callus formation for bone endochondral ossification (51). However, LSD1 is also reported to inhibit osteogenesis in other contexts. LSD1 represses BMP2 and WNT7B in osteoblasts preventing excess bone formation, and there is evidence that inhibiting LSD1 during certain stages of development can help increase bone mass in mice (52). Thus, LSD1 function strikes an important balance in osteogenesis, and the contextual regulatory factors that calibrate this balance warrant continued study.

### 3.3 Adipogenesis

In adipogenesis, LSD1 localizes to adipogenic promoters that display high levels of H3K9me2 to demethylate and promote differentiation of preadipocytes (53). As seen in myogenesis, LSD1 is sensitive to the metabolic state in regulating adipocyte function. In the fed state, LSD1 mediated demethylation of H3K9 promotes expression of PPARγ and adipogenesis (54). LSD1 is also critically important in the differentiation of brown adipocytes. Here, it suppresses the expression of mesenchymal stem cell maintenance genes during brown adipocyte differentiation by removing H3K4me2 marks in their protomoters (26). Further, LSD1, in complex with ZNF516 promotes the expression of brown adipocyte-specific promoters. Mice with LSD1 depleted in brown adipose tissue display impaired brown adipose development, with brown adipose that instead resembles white adipose (55).

Collectively, there is evidence that LSD1 plays important roles in promoting both the maintenance of mesenchymal stem cells and lineage-specific gene expression programs during mesenchymal differentiation. The molecular mechanisms that regulate how LSD1 responds to these different contexts, and how these contextual factors influence LSD1 function in sarcomas remain incompletely understood and require further study.

## 4 LSD1 in sarcomas

Despite the importance of LSD1 in various cellular aspects of mesenchymal development and the finding that LSD1 is overexpressed in many high-grade sarcomas, the role for LSD1 in many sarcomas has not been fully elucidated. Many of the sarcomas that have LSD1 overexpression are characterized by chromosomal translocations that result in the expression of fusion oncogenes. Most of the research on LSD1 has been done in fusion driven sarcomas, particularly in Ewing sarcoma and rhabdomyosarcoma. Below we discuss what is known about LSD1 function in sarcoma.

### 4.1 LSD1 in fusion sarcomas

#### 4.1.1 Ewing sarcoma

Ewing sarcoma is a common model system for studying LSD1, and has broad range of studies characterizing LSD1 as a molecular driver. Ewing sarcoma is defined as a small, round blue cell tumor that occurs in adolescents and young adults (1). The primary tumor frequently develops in long flat bones and is caused by the chromosomal translocation t(11;22)(q24;q12) in ~85% of cases (56, 57) (**Table 1**). This results in the expression of a fusion oncogene with an amino-terminal domain derived from the *EWSR1* gene on chromosome 11 and a carboxy-terminal domain derived from the *FLI1* gene on chromosome 22. The *EWSR* domain contains an intrinsically disordered domain that recruits transcriptional regulators (58), while the *FLI1* domain encodes an ETS family transcription factor (58). The resulting chimeric fusion protein, EWS/FLI, is pathognomonic to Ewing sarcoma, and acts as a powerful chromatin regulator and aberrant transcription factor by activating oncogenes and reshaping the epigenetic landscape to cause tumorigenesis through activity at Ewing-specific GGAA repeat response elements (59). As a transcription factor, EWS/FLI is difficult to target therapeutically, due to its lack of intrinsic enzymatic activity and its disordered nature (8). Instead, in recent years more effort has been placed on targeting associated epigenetic and transcriptional coregulators. On such factor is LSD1 which is overexpressed in Ewing sarcoma (13) (**Table 1**).

**Table 1:** Bone and Soft Tissue Sarcoma classifications and clinical trial status.

Tumor	Type	Fusion	LSD1 Expression	Clinical Trial Status
Ewing Sarcoma	Bone	EWS/FLI	Overexpressed	•Recruiting for Phase 1 Expansion SP-2577
				•Rollover for SP-2577
				•Recruiting for Phase 1 JBI-802
Rhabdomyosarcoma	Soft Tissue	PAX3/FOXO1	Overexpressed	•Recruiting for Phase 1 JBI-802
		PAX7/FOXO1		
Myxoid Liposarcoma	Soft Tissue	FUS/DDIT3	Potentially Overexpressed	•Recruiting for Phase 1 Expansion SP-2577
				•Rollover for SP-2577
				•Recruiting for Phase 1 JBI-802
Desmoplastic Small Round Cell Tumor	Soft Tissue	EWS/WT1	Overexpressed	•Recruiting for Phase 1 Expansion SP-2577
				•Rollover for SP-2577
				•Recruiting for Phase 1 JBI-802
Clear Cell Sarcoma	Soft Tissue	EWS/ATF1	Unknown	•Recruiting for Phase 1 Expansion SP-2577
				•Rollover for SP-2577
				•Recruiting for Phase 1 JBI-802
Synovial Sarcoma	Soft Tissue	SS18/SSX	Overexpressed	•Recruiting for Phase 1 JBI-802
Osteosarcoma	Bone	—	Overexpressed	•Recruiting for Phase 1 JBI-802
Chondrosarcoma	Soft Tissue	—	Overexpressed	•Recruiting for Phase 1 Expansion SP-2577
				•Rollover for SP-2577
				•Recruiting for Phase 1 JBI-802
Malignant Peripheral Neural Sheath Tumor	Soft Tissue	—	Overexpressed	•Recruiting for Phase 1 JBI-802

Every bone and soft tissue sarcoma discussed in this review broken down by type of sarcoma, fusion oncoprotein presence, the expression level of LSD1 in the sarcoma, and the ongoing clinical trials targeting LSD1 available for each sarcoma.

**Figure 2:**
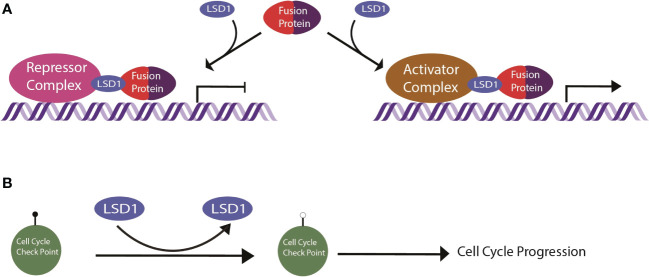
Schematic of LSD1 mechanism in sarcomas. **(A)** General mechanism of LSD1 in fusion driven sarcomas. The fusion protein recruits repressor or activator complexes *via* LSD1 targeting to enact transcriptional deregulation. **(B)** LSD1 in nonfusion sarcomas demethylates cell cycle check point proteins, inducing cell cycle progression.

EWS/FLI localizes to promoters, enhancers, and super enhancers throughout the genome and leads to genome-wide relocalization of LSD1 (60). EWS/FLI coopts the function of LSD1 by recruiting LSD1 complexes to EWS/FLI binding sites, altering the chromatin state, and disrupting related gene expression at those loci (60) (**Figure 2A**). For example, the N-terminal region of EWS/FLI recruits the LSD1-NuRD complex to repress transcription of tumor suppressor genes, promoting oncogenesis (58). This was demonstrated for the tumor suppressor genes *LOX* and *TGFBR2* by Sankar, et al. (61). Repression of *LOX* and *TGFBR2 via* LSD1-NuRD is dependent on EWS/FLI, suggesting the fusion oncoprotein actively recruits LSD1-NuRD to the specific regulatory loci for these genes (61). In a separate and distinct genetic repressive mechanism, LSD1 binds in super clusters at various enhancers in the absence of EWS/FLI, but once EWS/FLI is expressed, it colocalizes to these enhancers, destabilizes LSD1 binding to chromatin, and reduces downstream gene expression (60). Interestingly, there is also evidence suggesting another set of loci where EWS/FLI recruitment of LSD1 leads to gene activation (60). The details of the LSD1 mediated gene activation pathways remain unclear and are a topic of ongoing study (14, 60). LSD1 is thus a critical co-regulator of EWS/FLI, contributing to both gene activation and gene repression.

Genetic depletion and pharmacological blockade of LSD1 reverses both EWS/FLI-mediated gene activation and repression (14, 62). This stands in contrast to other epigenetic targeted therapies like histone deacetylase inhibitors that only target EWS/FLI-mediated repression (62). There is a body of evidence demonstrating that the transcriptional changes caused by treatment with SP-2509 (Salarius Pharmaceuticals) and SP-2577 (Salarius Pharmaceuticals) blocks cell growth and survival in Ewing sarcoma patient derived cell lines (61). Interestingly, treatment with these inhibitors shows only modest changes in histone methylation, primarily at H3K9 (62). Of note, H3K9me1 marks decrease with SP-2509 treatment while H3K9me2 and H3K9me3 increase with SP-2509 treatment (62). LSD1 is only able to demethylate mono- and di- methylated lysine residues, meaning another mechanism functions to increase trimethyl marks at H3K9. How treatment with these inhibitors impacts the epigenomic landscape and 3D chromatin architecture are areas of continued study.

**Figure 3:**
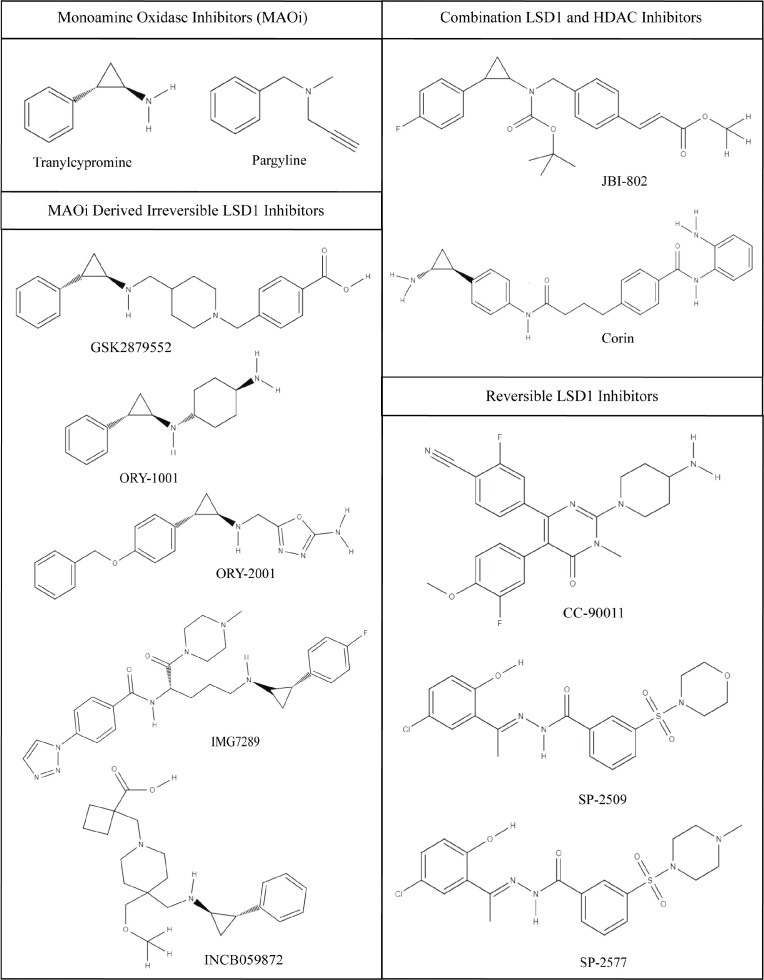
Chemical structures of LSD1 inhibitors. Monoamine Oxidase Inhibitors (MAOi); tranylcypromine and pargyline, tranylcypromine derivative irreversible LSD1 inhibitors; GSK2879552, ORY-1001, ORY-2002, IMG7289, INCB059872, combination LSD1 and HDAC inhibitors; JBI-802, Corin, and reversible LSD1 inhibitors; CC-90011, SP-2509, and SP-2577. Images were generated using MolView.

Notably, only small molecule blockade with the allosteric reversible inhibitors SP-2509 and SP-2577 show this activity (14, 63). Other LSD1 inhibitors, like ORY-1001 and GSK2879552 (**Figure 3**), are irreversible LSD1 inhibitors that covalently modify the FAD cofactor to block catalytic activity. Blocking LSD1 function through this binding mechanism does not have any notable activity against Ewing sarcoma cell growth (14, 63). Having been shown by multiple groups, this suggests that LSD1 catalytic function is not necessary for Ewing sarcoma growth and instead the critical role for LSD1 is nonenzymatic. Such nonenzymatic function has been described in other contexts like prostate cancer and AML (64, 65), but the specific nonenzymatic function for LSD1 in Ewing sarcoma is still unknown. SP-2509, and its clinical analog SP-2577, inhibit both the enzymatic and scaffolding functions of LSD1 and may explain their unique activity (62). As such, more work is required to understand nonenzymatic LSD1 function in Ewing sarcoma and how disruption of this function leads to anti-tumor efficacy, and these remain active areas of study.

#### 4.1.2 Rhabdomyosarcoma

Rhabdomyosarcoma (RMS) is another sarcoma classically driven by a fusion protein and exhibits strong LSD1 overexpression. RMS is the most common soft tissue sarcoma in children, and is subdivided into alveolar and embryonal rhabdomyosarcoma based on the histological makeup of the tumor (66). About 31% are alveolar and present with an alveolar pattern on histology, and 58% are embryonal with sheets and nests on histology, while the remaining 11% are made up of smaller subtypes with other histological patterns (66). The alveolar subtype of rhabdomyosarcoma is most often diagnosed by the presence of the fusion protein PAX3/FOXO1 from the chromosomal translocation t(2;13), or the less common translocation t(1;13) forming PAX7/FOXO1 (1, 66) (**Table 1**). PAX3 and PAX7 are members of the paired box family of transcription factors, and FOXO1 is a forkhead transcription factor (1). When fused together they create an aberrant oncogenic transcription factor pathognomonic to alveolar RMS and analogous to EWS/FLI in Ewing sarcoma (1). Generally, the embryonal RMS does not have an associated pathognomonic chromosomal translocation, and instead tends toward a higher genetic mutational burden while arising in older patients than the alveolar RMS subtype (67).

In the original LSD1 expression screen done by Schildhaus et al., RMS was one of the strongest hits, and both alveolar and embryonal subtypes had elevated LSD1 expression (12) (**Table 1**). Clinically, elevated LSD1 levels have been associated with poor prognosis in other cancer types, with evidence suggesting the same in RMS (68). However, to date there have been no papers looking into LSD1 function specifically in embryonal RMS subtypes. Instead the focus has been on LSD1 function in fusion positive alveolar RMS.

Like EWS/FLI of Ewing sarcoma, the PAX3/FOXO1 alters the function of other chromatin regulatory factors (**Figure 2A**). In 2016, Bohm et al. identified LSD1, CHD4, and RCOR1 in a dual proteomic and siRNA screen for critical interactors of PAX3/FOXO1 in alveolar RMS (69). Both LSD1 and CHD4 were shown to coimmunoprecipitate with the fusion (69). When looking at specific PAX3/FOXO1 target genes, LSD1 and CHD4 depletion had similar effects on the expression of myosin light chain 1 gene, *MYL1*, but opposite effects on *CDH3 (* 69). This suggests LSD1 may be present and functioning in different complexes at different targets. Additional studies are required to determine the suite of LSD1 complexes found in RMS cells. Follow up studies focused more on the role for CHD4, as LSD1 depletion showed minimal effect in short-term growth assays. That genetic depletion of LSD1 had limited effects in short-term proliferation was confirmed by Marques et al. in assays of RH4 cells (70). Similarly, an irreversible LSD1 enzymatic inhibitor, GSK690, did not induce cell death in other RMS cell lines in tissue culture (68).

Recent work has shown that LSD1 instead plays an important role in anchorage independent growth and adhesion in RMS (71). Knockdown of LSD1 in RMS cells reduces expression of extracellular matrix genes and decreases clonogenic growth, reducing tumor growth, effects that are not assayed in short term growth screens. Further investigation into the ability of RMS cell lines to resist LSD1 inhibitor treatment by Haydn et al., showed that where LSD1 inhibition alone is insufficient, LSD1 inhibition in combination with HDAC inhibitors synergistically induced cell death through the mitochondrial apoptotic pathway (68). Importantly, testing of the allosteric inhibitor SP-2577 by the Pediatric Preclinical Testing Consortium showed that RMS models were even more sensitive than the Ewing sarcoma models tested (72). Taken together, the relationship between LSD1 and PAX3/FOXO1 shows many parallels to LSD1 and EWS/FLI in Ewing sarcoma, supporting LSD1 as a promising therapeutic target in this disease. More work is required to better understand how LSD1 contributes to RMS biology and to elucidate the mechanism of action of SP-2577.

#### 4.1.3 Myxoid liposarcoma

While the most work on LSD1 as a molecular driver has been done in Ewing sarcoma and fusion positive RMS, there is some evidence supporting LSD1 as a driver in many other fusion sarcomas as well. These other model systems have not been studied as much, so the details of LSD1 function in these settings are even less understood. One such sarcoma is Mxyoid Liposarcoma, a lipogenic tumor that develops in deep soft tissues (73) (**Table 1**). It is usually a lower grade tumor made up of cells that are in various different stages of adipocyte differentiation together in a myxoid matrix (1). This sarcoma is most often characterized by the chromosomal translocation t(12;16) resulting in a chimeric fusion protein with domains from FUS and DDIT3/CHOP (73)(**Table 1**). LSD1 was overexpressed in 1 out of 5 tested myxoid liposarcoma tumor samples, suggesting minimal LSD1 overexpression (12) (**Table 1**). However, the small molecule reversible LSD1 inhibitor, SP-2577, still reduces cell viability in myxoid liposarcoma cell lines (15). Moreover, additional studies have shown that LSD1 coimmunoprecipitates with FUS/DDIT3 in myxoid liposarcoma cells, indicating the expression of the fusion protein may alter LSD1 localization and function even if there is less LSD1 overexpression (73) (**Figure 2A**). More studies are required to understand the role for LSD1 here and whether LSD1 function changes with the differentiation state of the tumor cells, but the early evidence suggests that these tumors are sensitive to allosteric LSD1 inhibition (15).

#### 4.1.4 Desmoplastic small round cell tumor

Desmoplastic small round cell tumors (DSRCT) are very rare and aggressive mesenchymal tumors. These sarcomas are generally characterized by expression of a EWS/WT1 fusion arising from the t(11;22)(p13;q12) chromosomal translocation. Interestingly, the EWS/WT1 transcriptional program bears some similarity to that driven by EWS/FLI, independent of those gene directly regulated by Ewing-specific GGAA response elements (74) (**Table 1**). This suggests that LSD1 may also be a critical coregulator of EWS/WT1 (**Figure 2A**), and that LSD1 blockade may similarly reverse the oncogenic gene expression patterns, but this requires additional study. DSRCT was a hit in the LSD1 expression screen by Schildhaus et al. further suggesting that LSD1 plays a significant role in this sarcoma (12) (**Table 1**). In addition, reversible LSD1 inhibitor, SP-2577, significantly impaired growth of patient-derived xenograft organoids and reduced time to event in *in vivo* PDX models of DSRCT (15). Like in Ewing sarcoma, nonenzymatic LSD1 activity is likely important in this tumor, as Romo-Morales et al., provide evidence that solely catalytic inhibition with irreversible inhibitors of LSD1 shows no antitumor activity (63).

#### 4.1.5 Clear cell sarcoma

Clear cell sarcoma is a malignant melanoma of the soft tissue, derived from neural crest cells and characterized by the expression of an EWS/ATF1 fusion protein derived from a **t(12;22)(q13;q12)** chromosomal translocation (75) (**Table 1**). Clear cell sarcoma was not tested in the LSD1 expression screen, so it is not certain whether clear cell sarcoma overexpresses LSD1 (**Table 1**). However, treating clear cell sarcoma cell lines with SP-2577 reduced cell viability suggesting that LSD1 may have an important role in this tumor (15). Unfortunately, there are no published studies looking specifically at LSD1 in clear cell sarcoma, and more work is done to understand the biological function of LSD1 here.

#### 4.1.6 Synovial sarcoma

Synovial sarcoma is either a pure spindle cell neoplasm or a combination of spindle cell and epithelioid neoplasm (1). Approximately 90% of synovial sarcomas have the t(x;18) chromosomal translocation, SS18/SSX, that combines the activation domain of SS18 and the repressor domain of SSX (1) (**Table 1**). Various domains in this fusion protein interact with Trithorax and polycomb protein complexes to regulate chromatin (76), or associate with BAF complex chromatin remodelers (5). Synovial sarcoma was found to overexpress LSD1in the overexpression screen discussed above (12) (**Table 1**). However, there is no other data further characterizing LSD1 function in this sarcoma.

Given the observed efficacy of allosteric LSD1 inhibitors in many sarcomas in addition to the elevated expression of LSD1, determining the nonenzymatic function of LSD1 in these fusion-driven tumors is critically important, especially as these inhibitors progress into the clinic.

### 4.2 LSD1 in nonfusion sarcomas

#### 4.2.1 Osteosarcoma

Unlike the tumors just discussed, there are other sarcomas, such as osteosarcoma, that are not associated with a chimeric pathognomonic fusion protein (77). Osteosarcoma is the most common primary malignant tumor of the bone that arises sporadically in adolescents and young adults (1). Instead of a chromosomal translocation, osteosarcoma generally contains multiple genetic mutations that activate various oncogenes and inactivate tumor suppressor genes such as p53 and Rb (77). These mutations disrupt cell cycle checkpoint restrictions, and lead to chromosomal instability and defects in DNA damage repair (77). However, like many of the fusion positive sarcomas, osteosarcoma was also a strong hit in the LSD1 expression screen performed by Bennani-Baiti (13) (**Table 1**).

LSD1 function in osteosarcomas is complex with many layers of regulation that are all disrupted when LSD1 is overexpressed. For example, in osteosarcoma LSD1 demethylates the p21 promoter, decreasing p21 expression and thus promoting cell cycle progression (78) (**Figure 2B**). USP22, an ubiquitin specific protease, stabilizes the LSD1/p21 interaction and facilitates the downregulated expression of p21. Furthermore, microRNA 140 inhibits the USP22 mediated LSD1 stabilization, which halts the cell cycle (78). Thus, excess LSD1 expression tips the balance toward promoting cell cycle progression (78). In another example, LATS1 is a tumor suppressor that is normally negatively regulated by LSD1 with long noncoding RNA, FOXP4-AS1 (79). LSD1 demethylates H3K4me2 and reduces LATS1 expression (79). However, in osteosarcoma both FOXP4-AS1 and LSD1 are overexpressed. Together, LATS1 is effectively silenced, promoting osteosarcoma progression (79). Additionally, SUV39H2, a histone lysine methyltransferase, is also overexpressed in osteosarcoma. Typically LSD1 and SUV39H2 coordinate the chromatin methylation signature to regulate e-cadherin expression, among other genes. In osteosarcoma, excess expression of these enzymes excessively downregulate e-cadherin, impairing cell adhesion and promoting a more aggressive mesenchymal phenotype (80).

In all of these cases, natural regulatory balances are disrupted by excess LSD1 and other overexpressed genes, contributing to oncogenesis. It is unclear whether overexpression of LSD1 in osteosarcoma is causal or a result of other upstream oncogenic events in osteosarcoma, though the latter seems more likely given the evidence to date. Even so, the overexpression of LSD1 is characteristic of osteosarcoma development and progression, and so LSD1 may be a useful target in this disease.

#### 4.2.2 Chondrosarcoma

Chondrosarcoma is another fusion negative sarcoma that overexpresses LSD1. Chondrosarcomas are a heterogeneous group of primary malignant tumors that arise from hyaline cartilage tissue (81). Unlike some of the other sarcomas already discussed, chondrosarcomas primarily affect adults, instead of pediatrics or adolescents and young adults (81). As a result chondrosarcoma patients tend to have a more favorable outcome as the tumor can be resected with better surgical margins (81). Chondrosarcomas are driven by a genetic mutation of isocitrate dehydrogenase (IDH) 1 and 2 in approximately 85% of cases (81). Another common epigenetic feature of these tumors is DNA hypermethylation of tumor suppressor genes p16 and RUNX3 (82). To date, there has been no specific research on LSD1 in chondrosarcoma apart from the LSD1 overexpression (13).

#### 4.2.3 Malignant peripheral neural sheath tumor

Malignant Peripheral Neural Sheath Tumor (MPNST) is an extremely rare sarcoma that also overexpresses LSD1. MPNSTs are the sixth most common soft tissue sarcoma, although it was only recognized by the WHO as its own classification of an aggressive neoplasm in 2020 (7). Most commonly MPNSTs arise in association with the genetic syndrome Neurofibromatosis 1, but sporadic cases also occur (5). Instead of a pathognomonic fusion protein, these tumors usually contain loss of function mutations in the SUZ12 and EED subunits of the histone modifying polycomb repressor complex 2 (PRC2) (5). PRC2 typically deposits di- and trimethyl marks on the H3 lysine 27 residue (H3K27me3) to repress associated genetic programs (5). These mutations occur frequently enough that loss of PRC2 and the subsequent loss of H3K27me3 marks are diagnostic markers for MPNST diagnosis (83). Interestingly, LSD1 cannot remove trimethyl marks on H3 lysine residues, and it does not target H3K27. Therefore the overexpression of LSD1 does not exacerbate the reduced methylation due to loss of the PCR proteins. As such, LSD1 likely plays an unknown role in MPNST development and more work is required to understand this role (12) (**Table 1**).

## 5 LSD1 in the clinic

Multiple classes of LSD1 inhibitor are currently in clinical development. These include irreversible inhibitors, reversible competitive inhibitors, and reversible noncompetitive inhibitors.

### 5.1 Irreversible inhibitors

The irreversible inhibitors are frequently derivatives of the monoamine oxidase inhibitor (MAOi), tranylcypromine (TCP; **Figure 3**). TCP was among the first described LSD1 inhibitors, due to the structural homology in the amine oxidase domain between MAO A/B and LSD1 (84). As a result TCP has relatively poor potency and specificity for LSD1. To increase the potency and specificity for LSD1, many TCP derivatives have additional chemical groups that improve binding interactions with the larger substrate binding pocket of LSD1. TCP and its derivatives form an irreversible covalent adduct with the FAD cofactor preventing demethylation (84).

Bennani-Baiti et al. demonstrated preclinical efficacy for TCP in some sarcoma model, but supraphysiologic concentrations of the drug were required to induce cell death due to the aforementioned issues with potency (13, 57). Thus, TCP is not clinically practical (57). Another MAOi, pargyline, was used to inhibit LSD1, but showed no improvement over TCP in specificity or kinetic profiles (85) (**Figure 3**). Many TCP derivatives remain in clinical trials for non-sarcoma malignancies. These derivatives include GSK2879552 (GlaxoSmithKline), ORY-1001/ORY-2001 (Oryzon Genomics), INCB059872 (Imago Biosciences), and IMG-7289 (Imago Biosciences). GSK2879552, underwent a phase 1 trial in acute myeloid leukemia (AML) (NCT02177812), but the risk vs benefit ratio, resulting from elevated adverse event rates, was not favorable enough to support further study (86). A similar result was obtained in small cell lung cancer (87).

Another TCP derived LSD1 inhibitor, ORY-1001 (Iadademstat), has been demonstrated to be safe in a phase 1 trial and is currently in phase 2 clinical trials (NCT05420636) in combination with paclitaxel, an antineoplastic drug, in small cell lung cancer and neuroendocrine carcinoma to determine response rate (88). Additionally, it is being studied in relapsed/refractory AML in combination with azacytidine, another epigenetic therapy targeting DNA methylation. This study is currently ongoing and showing a favorable ADME profile and high bioactivity in addition to a synergistic effect between the two drugs (89, 90). Its successor ORY-2001 is geared more towards CNS disorders treatment with its ability to cross the blood brain barrier and activity against both LSD1 and MAO-B (91). IMG-7289 is another irreversible LSD1 inhibitor that being investigated for in myelofibrosis (92) (NCT03136185) and essential thrombocythemia (93) (NCT04081220). The compound has been determined safe and has progressed into phase 2 studies, which are ongoing in both conditions (94). None of the above drugs have been studied in sarcomas.

Lee et al. discovered a more selective and potent LSD1 inhibitor, INCB059872, that has improved oral bioavailability (95). However, phase 1 clinical trials in relapsed or refractory Ewing sarcoma (NCT03514407) and phase 1/2 dose escalation clinical trials in several solid tumors in addition to Ewing sarcoma (NCT02712905) were all terminated for economic reasons (96, 97). Unfortunately no conclusions about its efficacy in sarcomas can be drawn from this study, and no successive studies are currently being planned (96, 97).

### 5.2 Reversible competitive inhibitors

Another class of LSD1 inhibitors developed are reversible competitive inhibitors that bind in the same pocket as the irreversible inhibitors, but do not form covalent adducts with the FAD cofactor. This blocks the binding of the histone H3 N-terminal tail substrate, but can dissociate to restore LSD1 activity. Kanouni et al. developed a strongly potent reversible LSD1 inhibitor, CC-90011, that induces terminal differentiation in AML and SCLC cancer cells (98) (**Figure 3**). Phase 1 clinical trials in advanced solid carcinomas and non-Hodgkin lymphoma (NCT02875223) are ongoing and the compound has expanded into multiple phase 1 trials in other cancers and in combination with other drugs (99). While CC-90011 shows some early promise in other cancer cell lines, no data has been collected from sarcoma cell lines or sarcoma tumors (98).

### 5.3 Reversible noncompetitive inhibitors

#### 5.3.1 Inhibitors in development

So far the most promising LSD1 inhibitors are the reversible noncompetitive inhibitors that reversibly bind LSD1 at a site on the protein that doesn’t block substrate binding. These inhibitors are also called allosteric and scaffolding inhibitors, and include SP-2509 and SP-2577 (64). SP-2509 was first described in 2013 (100). Sorna et al. performed a virtual structural based screen to identify novel selective and reversible compounds that noncompetitively inhibit LSD1 (100). SP-2509, has been extensively studied in Ewing sarcoma. In Ewing sarcoma cell lines SP-2509 disrupts transcriptional activity of the fusion protein, EWS-FLI, and leads to cell death (57). The concentrations required to induce cell death were much closer to clinically relevant levels below 1 µM than previous inhibitors (57). Furthermore, compared to Ewing sarcoma cells with shRNA knock down of EWS/FLI, wild type Ewing sarcoma cells with the EWS/FLI fusion oncoprotein were significantly more sensitive to SP-2509 treatment (57). Therefore, SP-2509 activity is thought to be context specific in Ewing sarcoma, in a manner related to LSD1 function as a coregulator of EWS/FLI (57).

The exact mechanism of SP-2509 is still unclear, however there is evidence to suggest it induces endoplasmic reticulum stress and the unfolded protein response, thus triggering caspase-3/7 activity and apoptosis (14). Differing from other LSD1 inhibitors, SP-2509 also inhibits certain protein-protein interactions and non-enzymatic functions of the enzyme. In a prostate cancer model system, SP-2509 treatment reduced the protein levels of LSD1 but not the RNA levels, which when treated with a protease inhibitor could be reversed (64). Furthermore, SP-2509 reduced the half-life of LSD1 in cells treated with cycloheximide to prevent any new LSD1 protein formation and was the only LSD1 inhibitor to block interactions between LSD1 and its interaction partner ZNF217 (64). Together this evidence strongly supports a mechanism where SP-2509 inhibits LSD1 not just by preventing catalysis, but also by inducing protein instability and subsequent degredation (64).

SP-2577 (seclidemstat) is the clinical analogue of SP-2509, with similar potency and improved solubility and oral bioavailability but reduced cell permeability (101). An *in vivo* evaluation of SP-2577 demonstrated growth inhibition in various Ewing sarcoma, rhabdomyosarcoma, and osteosarcoma models (72). In addition, levels of active transcriptional epigenetic marks, such as H3K4me2, were increased with SP-2577 treatment (72). Interestingly, in this Pediatric Preclinical Testing Consortium study tumor regression with SP-2577 treatment was only seen in one RMS model, so while the inhibitor prevents tumor progression, it could not shrink the current tumor as a single agent (72).

Even still, SP-2577 progressed to the clinic where safety and dose tolerability of the drug was determined in a broad range of advanced solid tumors (102). With a positive safety profile in advanced solid tumors, phase 1 trials investigating seclidemstat in relapsed or refractory Ewing sarcoma patients began in 2020 (103) (NCT03600649). This study aimed to determine safety and tolerability of the oral LSD1 inhibitor in addition to maximum and recommended doses for future clinical trials (103). Gastrointestinal adverse events were the first dose limiting toxicity, setting the maximum tolerated dose and recommended Phase 2 dose of seclidemstat at 900mg BID, thus confirming a positive safety profile of the compound (103). One patient showed target lesion shrinkage by the end of the second cycle, while two others had stable disease after the second cycle (103). There are 10 patients who benefited from seclidemstat in this trial and continue to use the medication as a part of a rollover protocol (104) (NCT05266196). These results supported the expansion into phase 2 clinical trials in both Ewing sarcoma and other Ewing-like tumors with other *EWSR1* gene fusions, including myxoid liposarcoma, DSRCT, and clear cell sarcoma (105) (NCT03600649), which is currently still recruiting patients. To date, SP-2577 is the most promising small molecule LSD1 inhibitor for sarcoma therapy.

#### 5.3.2 Resistance to noncompetitive inhibitors

Despite the promising data for reversible noncompetitive LSD1 inhibitors, cancer treatment as a single agent is often insufficient due to resistance development. Drug targets can accumulate mutations that render it resistant to the therapeutic drug. LSD1 in general has not been shown to gather somatic mutations, which is promising, but mutations are not the only method cancer cells can become resistant. Both Pishas et al. and Tokarsky et al. provided evidence that resistance to SP-2509 is modulated through mitochondrial dysfunction (14, 106). Interestingly, both studies showed that as Ewing sarcoma cells gain resistance to LSD1 inhibition, they adopt a less oncogenic phenotype (14, 106). Pishas et al. further found that resistance was relatively durable, even after removal of the drug, and suggested epigenetic reprogramming may play a role in the development of resistance (14). One way to reduce the incidence of drug resistance is combination therapy.

#### 5.3.3 Possible combinations therapies

Combination therapy was first studied in RMS since irreversible LSD1 inhibitors were not sufficient to kill RMS cells alone. The MAOi tranylcypromine derivative GSK690 did not induce cell death in RMS cell lines *in vitro*, but when combined with a histone deacetylase (HDAC) inhibitor, cell death was observed in several cell lines (68). The activities of both inhibitors was synergistic and led to increased mitochondrial apoptosis (68). This synergistic effect is also seen in acute myeloid lymphoma cell lines (107).

LSD1 inhibition is also likely to be used in combination with conventional chemotherapeutic agents. *In vitro* studies assaying the synergistic effect of LSD1 inhibitors with other chemotherapeutic agents in Ewing sarcoma showed synergy with cyclophosphamide, a DNA alkylating agent, and topotecan, a topoisomerase II inhibitor (105). Interestingly, another study showed that treatment with first line treatment for Ewing sarcoma sensitized the cells to both LSD1 and HDAC inhibition (108).

Instead of combining two separate compounds, another method to develop combination targeted therapy is to design a compound that intrinsically has multiple targets. JBI-802 is a single compound with activity against both LSD1 and HDAC6 (109). Sivanandhan et al. designed this novel dual inhibitor using computational chemistry approaches and demonstrated its efficacy *in vitro* against sarcomas and other hematological cancers (110) (**Table 1**). Another example is Corin, a synthetic compound that inhibits both HDAC1 and LSD1 in the CoREST complex (111). Corin shows preclinical efficacy in melanoma and squamous cell carcinoma cell lines, but has not been tested in sarcomas (111). More preclinical studies are needed to fully evaluate the potential of dual target compounds and to find therapeutic combinations that best pair with LSD1 inhibition. This work is needed to better understand how to include LSD1 inhibition into the standard of care to improve patient outcomes in sarcoma.

## 6 Conclusion

In this review we presented what is known about LSD1 structure, function, and interaction partners, in addition to how accumulation of LSD1 is a critical oncogenic driver in bone and soft tissue sarcomas. LSD1 is a lysine specific histone demethylase that removes mono and dimethyl marks on the H3 histone tail at H3K4 and H3K9. Due to reaction mechanism constraints, LSD1 is unable to demethylate trimethyl marks, but manipulation of LSD1 levels affects global tri-methyl levels, suggesting LSD1 is still important in regulating all types of methylation marks. LSD1 contains an amine oxidase domain and proceeds through reaction mechanism similar to monoamine oxidases. LSD1 mainly acts in CoREST or MTA complexes, but there is plenty of evidence showing it is incorporated in other complexes as well to demethylate histone and non-histone substrates *via* enzymatic mechanisms. Notably, recent studies suggest that LSD1 also possesses nonenzymatic function, though this is not well understood. As a result, LSD1 can repress and activate genetic programs through various mechanisms, pointing to the dynamic nature of LSD1.

LSD1 is overexpressed in numerous sarcomas supporting its role as an oncogenic driver. Ewing sarcoma and RMS are the most thoroughly characterized systems, as LSD1 has not been studied as much in the other discussed sarcomas. Determining mechanistically how LSD1 is driving oncogenesis would have significant implications in how to best target LSD1 clinically. Various irreversible and reversible competitive as well as reversible noncompetitive inhibitors have been tested, but so far the small molecule reversible noncompetitive inhibitors, SP-2509/SP-2577 are the most promising. However, the specific targeted function of LSD1 and the exact binding and mechanism of the small molecular inhibitor remain unclear. Even so, together the evidence supports LSD1 as a critical driver in bone and soft tissue sarcoma development, and a promising target for new therapeutics.

## Author contributions

RD and ET contributed to conception, writing, and editing of this manuscript. All authors contributed to the article and approved the submitted version.
